# Anomaly originated flexor digitorum superficialis muscle of the small finger: A case report

**DOI:** 10.1097/MD.0000000000034566

**Published:** 2023-08-04

**Authors:** Young-Keun Lee

**Affiliations:** a Department of Orthopedic Surgery, Research Institute of Clinical Medicine of Jeonbuk National University, Biomedical Research Institute of Jeonbuk National University Hospital, Jeonju, Jeonbuk, Republic of Korea.

**Keywords:** flexor digitorum superficialis, muscle anomaly, small finger

## Abstract

We present a clinical case of flexor digitorum superficialis (FDS) muscle belly of the small finger originating from the palm of a patient undergoing carpal tunnel surgery with a literature review.

**Patients concern:**

A 28-year-old right-handed woman visited our hospital with a chief complaint of a continuous tingling sensation and weakness in the right hand, which began after the volar side of her wrist was crushed by a machine during work 2 weeks prior. The patient complained of a continuous tingling sensation in the thumb, index, and middle fingers. The patient had a positive result on Tinel test of the median nerve of the wrist. As electromyography and nerve conduction velocities showed signs of severe injury in the right median nerve, exploration and carpal tunnel release were planned.

**Diagnosis:**

Carpal tunnel release was performed under regional anesthesia using the classical open approach. The median nerve in the distal forearm and distal portion of the flexor retinaculum appeared to be narrowed and compressed. An anomalous muscle originating from the flexor retinaculum is also observed.

**Intervention:**

The FDS muscle of the small finger was excised at the flexor retinaculum and musculotendinous junction and sutured to the flexor digitorum profundus tendon.

**Outcome:**

At the 37-month follow-up, the patient did not experience any tingling sensation or weakness. She showed excellent range of motion of the right small finger. The grip strength was 20 kg on both the right and left sides. Quick disabilities of the arm, shoulder, and hand score was 2.3.

**Conclusion:**

Asymptomatic small finger FDS muscle anomalies can occur, as demonstrated in this case study. Thus, physicians should familiarize themselves with small finger FDS muscle anomalies during interactions with patients to facilitate future treatments of patient complaints related to the hand, as well as wrist laceration or trauma requiring hand exploration.

## 1. Introduction

Various anomalies of the flexor digitorum superficialis (FDS) tendon and muscles of the small finger have been reported in the literature.^[1–3]^ In particular, there is an ongoing debate on whether anomalies of the FDS muscle are of single (either antebrachial or palmar) or dual (both antebrachial and palmar) origin.^[3,4]^ However, in the case of dual or palmar origin, an anomalous palmar muscle belly of the FDS muscle might be observed as a rare variation as a consequence of mismigration of its palmar anlage to the forearm.^[5]^ While there has been one study reported an anatomical origin (palmar) of the FDS muscle of the small finger, no clinical study exists to date.^[5]^ Here, we present a clinical case of an FDS muscle belly of the small finger originating from the palm of a patient undergoing carpal tunnel surgery, along with a literature review.

### 1.2. Consent

The patient signed an informed consent form for the publication of this case report and any accompanying images. The ethical approval for this study was waived by the ethics committee of Jeonbuk National University Hospital because it was a case report with fewer than 3 patients (2021-12-045).

## 2. Case presentation

A 28-year-old right-handed woman visited our hospital with a chief complaint of a continuous tingling sensation and weakness in the right hand, which began after the volar side of her wrist was crushed by a machine 2 weeks prior. As a homemaker, the patient had not performed laborious work prior to taking a job due to personal circumstances a month ago, which involved machines at a factory (8 hours per day, 5 days per week).

Upon physical examination, the volar and dorsal aspects of her right wrist appeared to have been crushed by the machine. However, severe swelling was not observed. The patient complained of a continuous tingling sensation in the thumb, index, and middle fingers. Range of motion of the fingers was normal. The patient showed a positive result on Tinel test for the median nerve in the wrist: grip strength of 5 kg on the right side and 10 kg on the left side; key pinch strength of 1 kg on both right and left sides; and quick disabilities of the arm, shoulder, and hand score of 63.^[6]^ As electromyography and nerve conduction velocities showed signs of severe injury to the right median nerve, exploration and carpal tunnel release were planned.

**Figure 1.**
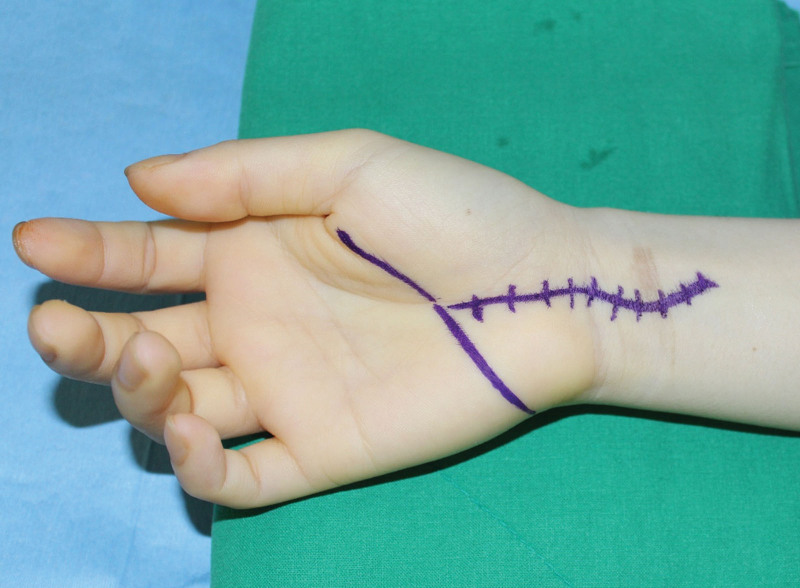
Initial preoperative photograph of the right hand and wrist of a 28-year-old female patient.

**Figure 2.**
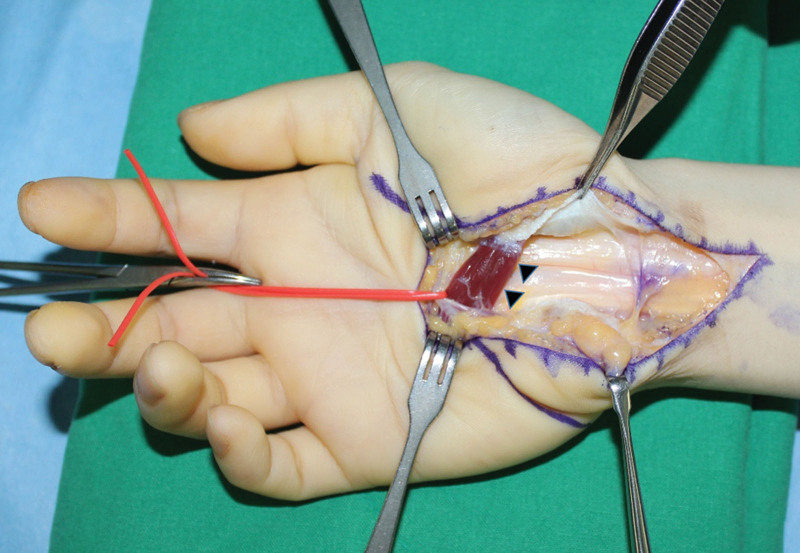
Intraoperative photograph of the right hand taken after incision in the deep transverse carpal ligament showing muscle anomalously originating from the flexor retinaculum (arrowheads).

**Figure 3.**
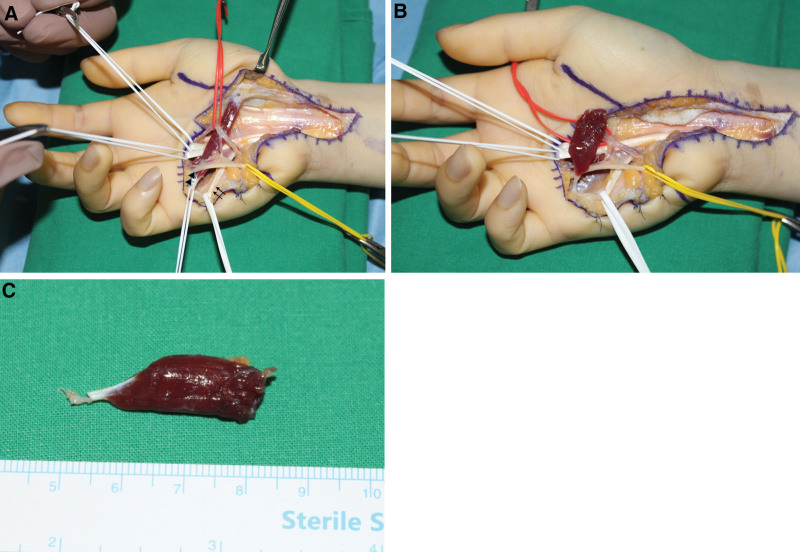
(A–C) Intraoperative photographs taken after performing additional exploration to the MP joint of the small finger, showing that the anomalously originating muscle runs parallel to the FDP tendon (arrows) with only 1 strand tendon (arrowheads) at the distal part (A). Photograph of the anomalously originating muscle excised from the flexor retinaculum (B) and the musculotendinous junction (C) are shown. FDP = flexor digitorum profundus, MP = metacarpophalangeal.

**Figure 4.**
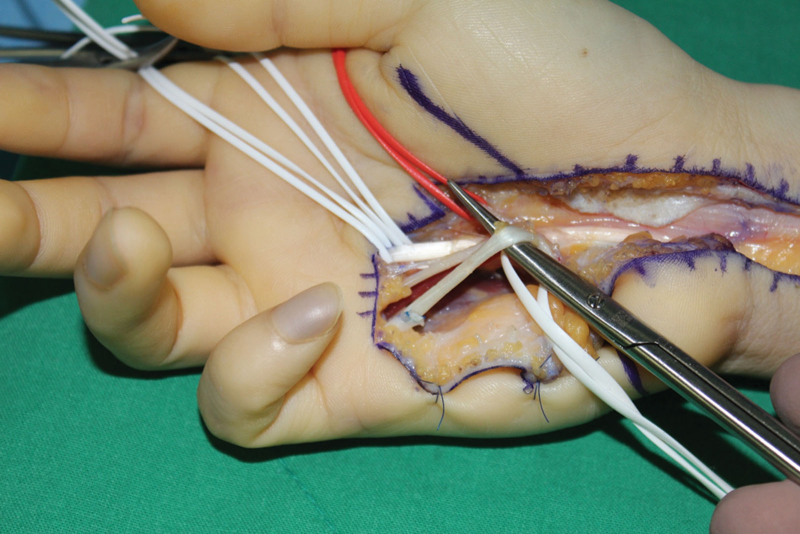
Intraoperative photograph showing the anomalous muscle being excised and the tendon being sutured to the FDP tendon. FDP = flexor digitorum profundus.

Carpal tunnel release was performed under regional anesthesia using the classical open approach (Fig. 1). The median nerve in the distal forearm and distal portion of the flexor retinaculum appeared to be narrowed and compressed. Anomalous muscle originating from the flexor retinaculum was also observed (Fig. 2). Further exploration was performed on the metacarpophalangeal joint along the muscle. As pulling of the tendon connected to this muscle resulted in flexion of the proximal interphalangeal joint of the small finger, we identified the FDS muscle of the small finger. The length and width of the muscles were 26 and 9 mm, respectively. The tendon portion is a single band parallel to the flexor digitorum profundus (FDP) tendon of the small finger. No connection was observed between the FDS and FDP tendons (Fig. 3A–C). Furthermore, a normal antebrachial FDS tendon running to the small finger within the carpal tunnel was not found. Additional exploration of the proximal interphalangeal joint and forearm was not performed. The FDS muscle of the small finger was excised at the flexor retinaculum and musculotendinous junction and sutured to the FDP tendon (Fig. 4).

**Figure 5.**
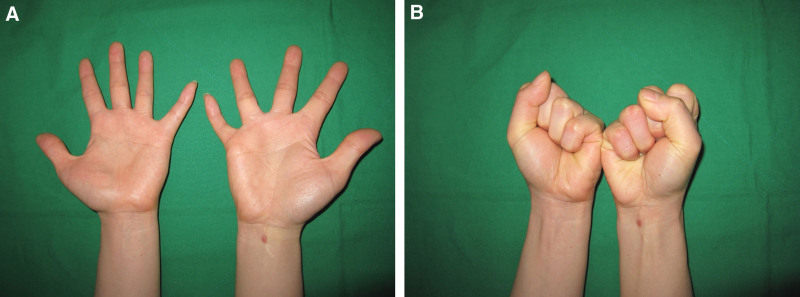
(A and B) Photographs obtained at the final follow-up (37 months after surgery) showing normal ROM of the small finger in the right hand. ROM = range of motion.

At the 37-month follow-up, the patient did not have any tingling sensations or weakness. The patient showed excellent range of motion of the right small finger. The grip strength was 20 kg on both the right and left sides. The quick disabilities of the arm, shoulder, and hand score was 2.3 (Fig. 5A and B).

## 3. Discussion

In general, experienced hand surgeons often view the FDS tendon of the small finger as thinner and functionally inferior to that of the other fingers. They found it difficult to locate the laceration during deep laceration treatments at the wrist.^[7,8]^ This has been supported by studies of various anomalies of the FDS tendon of the small finger.^[1,2,5,7–9]^ Baker et al^[7]^ analyzed the functional status of the FDS muscle of the small finger in 204 relatively healthy individuals and found that 16% of them showed absolutely deficient superficialis muscle function. They suggested that flexor profundus injury in the small finger should be treated as a major tendon injury, as it often results in greater morbidity than isolated profundus injuries in other fingers.

Reports of anomalies of the FDS muscle of the small finger are rare. Elliot et al^[10]^ presented 3 cases in which patients with FDS muscle anomalies complained of median nerve compression symptoms and palm swelling. These patients had FDS muscle anomalies in the index and long fingers. Their study also discussed the normal development of the FDS muscle from amphibians to humans and classified the FDS muscle anomalies in humans into 5 types.^[10]^ As in our case, the flexor retinaculum or an anomaly originating at the palmar level was classified as having a type II anomaly. However, in type II anomalies, the muscle originating from the flexor retinaculum is inserted into the long superficialis tendon, which starts from the forearm. As the long superficialis tendon did not exist in our case, it was thought to be a new type of anomaly not belonging to type II, or remaining in the form observed in amphibians.

In studies that reported superficialis muscle origin anomalies in the flexor retinaculum, all anomalies were observed in the index finger without causing clinical symptoms, such as mass and median nerve entrapment in the palm.^[11–13]^ Therefore, although most FDS muscle anomalies are known to have no clinical significance, clinicians should consider that palmar anomalies can clinically present with painless or painful palmar masses or carpal tunnel syndrome. No clinical symptoms were observed in this case. It might be due to the fact that the muscle belly was located on the outside of the carpal tunnel, and that the patient was a homemaker who did not engage in heavy labor without a history of trauma in the palm. Kobayashi et al^[5]^ reported an anomaly in the FDS muscle of the small finger, similar to our case, in an 80-year-old female cadaver in which the antebrachial FDS tendon was absent. However, unlike the single tendon from the FDS muscle in our case, Kobayashi et al^[5]^ found that the FDS tendon bifurcated between which the FDP tendon was transmitted. In addition, their study suggested that despite the fact that it was not clinically or anatomically well analyzed, asymptomatic FDS muscle anomalies in the small finger could occur frequently.

The limitation of this study was that, as a cadaver was not involved, we could not dissect beyond the forearm to obtain more accurate findings.

## 4. Conclusion

Asymptomatic small finger FDS muscle anomalies are likely to occur, as demonstrated in this case study. Thus, physicians should familiarize themselves with small finger FDS muscle anomalies during interactions with patients to facilitate future treatments of patient complaints related to the hand, as well as wrist laceration or trauma requiring hand exploration.

## Author contributions

**Conceptualization:** Young-Keun Lee.

**Data curation:** Young-Keun Lee.

**Formal analysis:** Young-Keun Lee.

**Methodology:** Young-Keun Lee.

**Software:** Young-Keun Lee.

**Writing – original draft:** Young-Keun Lee.

**Writing – review & editing:** Young-Keun Lee.
